# Implementation of an Intrahospital Transport Checklist for Emergency Department Admissions to Intensive Care

**DOI:** 10.1097/pq9.0000000000000426

**Published:** 2021-06-23

**Authors:** April M.-R. Venn, Cecilia A. Sotomayor, Sandip A. Godambe, Turaj Vazifedan, Andrea D. Jennings, Faiqa A. Qureshi, Paul C. Mullan

**Affiliations:** From the * Children’s Hospital of the King’s Daughters; † Department of Pediatrics, Eastern Virginia Medical School; ‡ Department of Emergency Medicine, Columbia University Vagelos College of Physicians and Surgeons, New York Presbyterian Hospital; § Division of Emergency Medicine, Department of Pediatrics, Eastern Virginia Medical School

## Abstract

**Introduction:**

Intrahospital transports (IHTs) are high-risk activities with the potential for adverse outcomes. Suboptimal care of a patient in our emergency department (ED) needing IHT to the pediatric intensive care unit (ICU) identified improvement opportunities. We describe implementing a novel checklist (Briefing ED-to-ICU Transport To Exit Ready: BETTER) for improving the IHT safety of pediatric ED patients admitted to the pediatric ICU.

**Methods:**

A multidisciplinary team used the Model for Improvement to create a key driver diagram and process map. An evidence-based IHT checklist was implemented on July 23, 2019 after multiple plan-do-study-act checklist revisions. The specific aim was a ≥80% checklist completion rate for 6 months and maintaining that rate for 6 months. An anonymous, voluntary survey of ED nurses and physicians, 9 months postimplementation, evaluated perceived improvements in IHT safety. The outcome measure was the proportion of IHT-related incident reports, per ED-to-pediatric ICU admission, comparing baseline (2-year preimplementation) and intervention (1-year postimplementation) periods. Balancing measures included a quantitative assessment for any throughput measure delays and a survey question on perceived delays.

**Results:**

From July 23, 2019 to July 22, 2020, 335 (84%) of 400 ED-to-ICU admissions had completed IHT checklists. Ninety percent of survey respondents (84% response rate) agreed that the checklist improved IHT safety. The incident report rate was lower in the intervention period (0.5% versus 2.3%; *P* = 0.03), with special cause improvement on T-chart analysis. Balancing measures did not indicate any delays secondary to checklist implementation.

**Conclusions:**

This IHT checklist was feasible and associated with improvements in perceived safety and incident event reporting. Further studies are needed to assess generalizability.

## Abstract 2

Supplemental Digital Content is available in the text.

## INTRODUCTION

Emergency department (ED) patients admitted to the intensive care unit (ICU) require safe intrahospital transport (IHT). During the IHT process, patients can experience adverse events from physiologic deterioration, equipment issues, or medication issues. Prior studies report unexpected adverse events or physiological derangements during IHT from the ED and ICU settings as high as 75% of the time.^1,2^ In one study, physiologic deterioration occurred in 72% of IHTs, with therapeutic interventions required in 14% of these events.^2^ Adverse events attributed to equipment issues are another common issue and occur during 8%–46% of IHT of critically ill patients.^1–5^

In general EDs, the implementation of IHT checklists has been associated with reductions in adverse events.^4,6–9^ Checklists have been used in the pediatric ED setting to improve patient safety.^10^ However, we are unaware of any checklists to improve IHT safety for pediatric ED patients admitted to the pediatric ICU.

Following an adverse event involving an intubated pediatric patient during IHT to our PICU, the hospital safety leadership conducted a root cause analysis. The transport team did not have enough medications to keep the patient fully sedated during the longer than expected transport time to the PICU. The root cause analysis team identified several issues, including inadequate team-based assessment before transport and insufficient preparation of medication and equipment, as contributing factors to the event. The ED formed a multidisciplinary quality improvement (QI) team, composed of nurses and physicians with QI experience, to design an intervention to address IHT safety proactively using the Model for Improvement framework. This QI team used evidence-based recommendations to create the *B*riefing *E*D*-to*-ICU *T*ransport *T*o *E*xit *R*eady (BETTER) Checklist. The BETTER checklist’s global aim was to improve IHT safety for ED patients admitted to the PICU.

## METHODS

### Setting and Study Design

This study occurred in a 31-bed Level I pediatric trauma ED in a freestanding academic children’s hospital in the United States. In 2019, there were 56,231 ED visits, with a 0.7% medical PICU admission rate. The ED is located on the ground floor, and the PICU is located on the third floor. A walk up to the PICU from the ED, without a patient or any equipment, takes approximately 3–4 minutes. Sixteen pediatric emergency medicine (PEM) attending physicians who supervise resident physicians and PEM fellows staff the ED.^11,12^ The institutional review board approved this study.

### Subjects

Only medical PICU patients were eligible for inclusion during the baseline (July 23, 2018–July 22, 2019) and intervention periods (July 23, 2019–July 22, 2020). The team did not mandate the use of the checklist for surgical PICU admissions. PEM physicians and ED nurses were eligible for the April 2020 survey if they worked full-time (>0.6 full-time-equivalent) in our ED.

#### Development of IHT Checklist

**Fig. 1.**
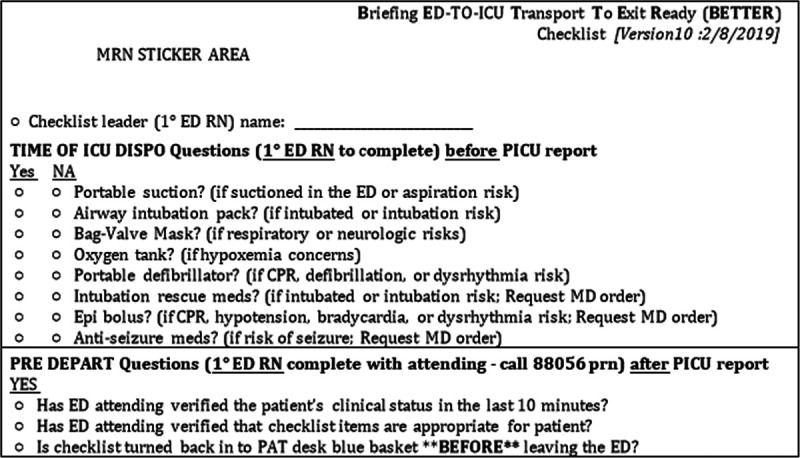
The implemented version of the IHT checklist (BETTER) for patients admitted from the ED to the ICU. CPR, cardiopulmonary resuscitation; DISPO, disposition; MD, physician; MRN, medical record number; PAT, patient access technician, ie, ED secretary; RN, nurse.

To ensure face and content validity in its design, we incorporated components of the initial checklist identified as high-risk issues based on the published IHT event and human factors literature, local experiences with IHT safety issues, and feedback from multidisciplinary ED stakeholders.^3,5,6,12–18^ The QI team did not aim to cover every aspect of IHT care. Instead, checklist items served as a cognitive aid to enhance a team member’s situational awareness on areas susceptible to omission during clinical practice.^19^ Checklist items covered issues related to equipment, medication, clinical assessment, and team communication. The team designed the checklist tool to be paper-based, self-explanatory, read-do checklist format, adaptive, and take less than 2 minutes to complete.^20^ QI team members used the pilot version of the checklist on 15 patients during the baseline period (December 3, 2018–April 11, 2019). They made 9 iterative changes to the pilot version of the checklist, based on plan-do-study-act cycles with frontline user feedback, before settling on the final checklist version (Fig. 1).

**Fig. 2.**
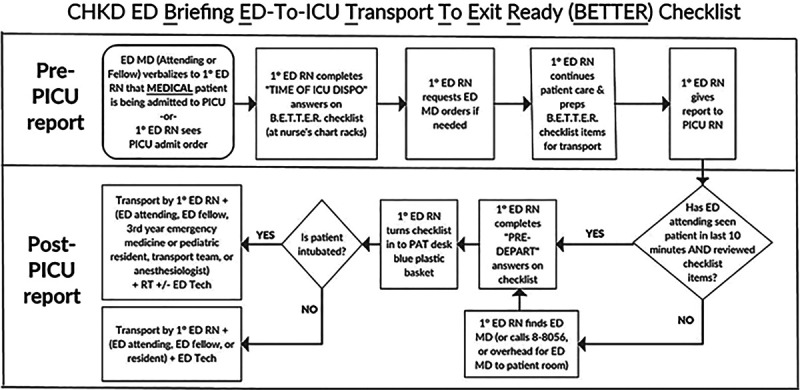
Process map for using the intrahospital transport checklist for patients admitted from the ED to the ICU. DISPO, disposition; MD, physician; PAT, patient access technician, ie, ED secretary; RN, nurse; RT, respiratory therapist.

The feedback from the piloting teams helped to design a process map to standardize the process for using the final checklist version (Fig. 2). The process started with the patient’s primary ED nurse completing the top half of the checklist after the ED attending determined that the patient had a PICU disposition. Once the PICU was ready, the ED nurse would phone the PICU nurse to give the nursing report. The ED nurse would then find the ED attending for a team huddle and ensure that 4 activities had occurred. First, the ED nurse would review the checked off items with the ED attending to anticipate any patient care issues that might arise during the IHT process; if additional issues or items were discussed during this team huddle, the nurse ensured that these items were prepared for the patient (Fig. 2, post-PICU report, first diamond-shaped decision). Second, the ED nurse would ensure that the ED attending had examined the patient at the bedside, within the prior 10 minutes, to confirm that the patient was clinically stable for IHT. Third, the nurse would ensure that the right team members transported the patient according to the guidelines described on the process map.^1,21^ Last, the ED nurse would return the completed checklist to a basket at the ED secretary’s desk immediately before ED departure. The QI team did not mandate the use of the checklist for surgical PICU admissions after they learned, during the checklist trials, that the surgical team members had often departed the ED before the time in which the team huddle would take place. QI team members collected completed checklists and entered data into a Microsoft Excel (Redmond, Wash.) database.

**Fig. 3.**
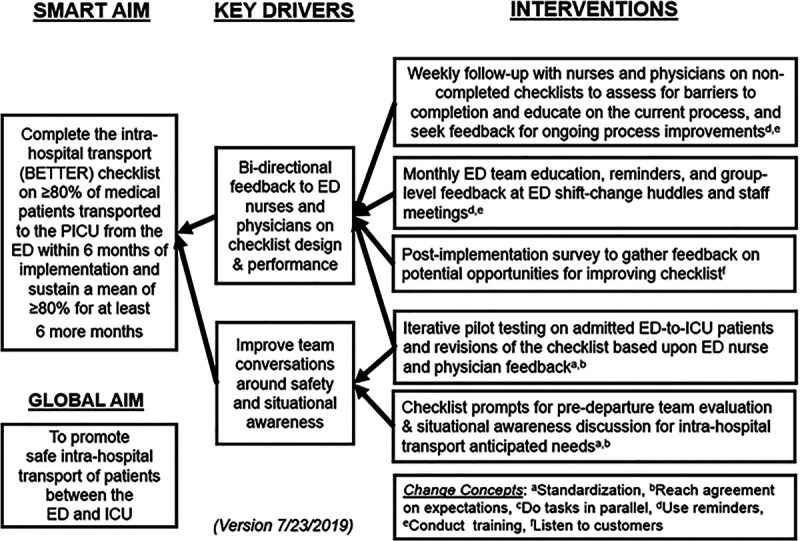
Key driver diagram.

ED nursing leadership educated all team members twice daily at 7:00 am and 7:00 pm ED team shift huddles, with a 2-minute overview on appropriate checklist usage, during the week before the implementation date (July 23, 2019). ED physicians learned about the new IHT checklist at these shift huddles and during physician meetings. QI team members followed up individually with any ED nurses or physicians who did not receive education in these forums. All eligible ED patients admitted to the PICU were identified in a newly created, validated report. A QI team member followed up with the patient’s primary nurse to identify barriers to checklist usage for patients without a checklist. The team posted the monthly completion rate of BETTER checklists on a staff bulletin board and reviewed performance at monthly staff meetings. These implementation components encompassed the changes and primary drivers of our project’s key driver diagram (Fig. 3).

#### Development of Checklist Perception Survey

The QI team created a novel survey to determine the BETTER checklist’s perceived impact, based on 3 quality domains.^22,23^ The first domain focused on safety, including changes in team communication and bedside assessment frequency. The second domain assessed team situational awareness, including changes in team perception of potential adverse events and team behavior changes related to anticipating such events. The third domain addressed the checklist’s operational value, assessing for any unintended harmful delays and determining the checklist’s perceived value. The QI team initially created at least 4 question items for each domain.^22^

Four ED nurses and 3 ED physicians reviewed the initial survey draft for face and content validity and offered feedback. Based on this feedback, the QI team made survey changes based on the group’s majority consensus. Each group member individually completed a clinical sensibility tool, and the QI team made minor modifications based on these results. Five senior emergency medicine residents pilot-tested the final survey for usability to assess the survey’s clarity and completion time. The final survey had 7 statements related to the 3 domains (with 5-level Likert scales), 3 demographic questions, and 1 open-ended feedback question (**Figure 1. Supplemental Digital Content 1,** http://links.lww.com/PQ9/A269). This paper-based survey was anonymous and voluntary. The QI team provided no incentives for survey completion. ED charge nurses or QI team members distributed surveys in paper format at the twice-daily shift huddles for 1 week in April 2020 (9 months postimplementation). Later on in their shift, respondents turned in surveys into a large envelope taped to a wall in the ED’s shared charting area.

### Outcomes and Analysis

#### Transport Checklist

The specific, measurable, attainable, relevant, time-bound (SMART) aim was a process measure to have ED staff complete ≥80% of BETTER checklists within 6 months of implementation and sustain a mean of ≥80% completion for at least 6 months.^6,7,14^ We defined “used” as having marked at least 1 checklist item and “completed” as having 100% of the checklist items marked.^24^ If a checklist item was blank (unchecked), it was considered not performed (eg, if either of the last 2 items related to the team huddle were left blank), and the checklist was not counted as “completed.” A run chart displayed the proportions of completed checklists over time.

One balancing measure was the time interval between 2 time-stamps in the electronic medical record: the physician’s order to admit the patient to the PICU and the patient’s departure time from the ED. Mean values between baseline and intervention periods were compared using a t-Test. Data analyses were performed with Minitab v19.0 (State College, Pa.).

For the outcome measure, the QI team queried the hospital’s safety reporting system database to determine the occurrence of all issues related to IHT.^3^ To determine the query terms, a Pubmed MESH term search was performed for “intra-hospital transport” and “transportation of patients.” The following terms resulted: transport, transportation, stretcher, transfer, and transition. By consensus, the study team added 2 additional terms: elevator and wheelchair. A query searched these 7 terms in our database to create an initial report. Two study authors (P.C.M. and S.A.G.) blinded themselves to event dates and independently excluded anything in the initial report that did not relate to a medical patient’s IHT issue between the ED and the PICU. Each included event was classified according to the severity of harm using an accepted PEM classification system.^25^ Discrepancies were discussed until a consensus was reached. The outcome measure was a comparison, between the baseline and interventions periods, of the proportion of these ED-to-PICU patients with IHT-related incident reports divided by the number of medical PICU admissions during each period, using a 2-sample test of proportions; also, a T chart identified any special cause variation in the number of days between incident reports. The hospital-wide, overall monthly incident reporting rates for all safety events were compared between baseline and intervention periods with a 2-sample *t* test.

#### Survey

The survey’s primary aim was that a consensus majority (≥80%) of both nurses and physicians would agree (“agree” or “strongly agree”) that “the BETTER checklist improves the safety of transporting patients to the PICU.”^26^ The Mann–Whitney test analyzed for any differences in responses between the ED nurse and PEM physician groups. Respondents who had not been working at least 6 months before the checklist implementation had their pre-post perception question answers (questions 5a/5b/5c) excluded. Spearman’s rank correlation measured the associations between the frequency of reported checklist usage and the survey questions 5a, 5b, and 5c. The balancing measure question for the survey was whether “the time to complete the BETTER checklist did not add significant delays in transport”; the QI team hypothesized that a consensus majority (≥80%) would not disagree.^27^ Free-text comments in the final question were transcribed verbatim. A *P* < 0.05 was considered statically significant for any checklist or survey outcomes.

## RESULTS

**Fig. 4.**
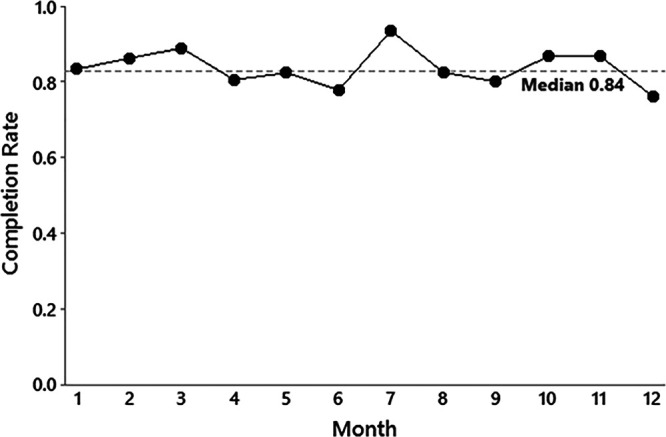
Run chart of the proportion of completed checklists during the intervention period for the IHT of patients admitted to the ICU from the ED.

Between July 23, 2019 and July 22, 2020, 373 (93%) of the 400 medical PICU admits had a BETTER checklist used, and 335 (84%) of these checklists were considered complete. This completion rate achieved our primary outcome and SMART aim for the project (Fig. 4), with mean completion rates of 83% and 85% in the first and second 6-month periods, respectively. Of the checklist items that had a response marked, the most commonly needed equipment item was an oxygen tank (80%), followed by a bag-valve-mask device (70%), portable suction (49%), airway intubation pack (18%), and a portable defibrillator (3%); the most common medications were intubation rescue medications (9%), followed by antiepileptics (7%) and epinephrine (4%). Of the completed checklists, a median of 2 [interquartile range (IQR) 1, 3] equipment and/or medication items were marked as necessary for IHT. Of the used checklists, items were skipped (ie, “blank”) in 2%–3% of equipment items, 2% of medication items, and 5% of ED attending verification items. There was no difference in our balancing measure between the baseline and intervention periods with mean intervals of 72.1 and 76.8 minutes, respectively (Difference = 4.7 minutes, 95% confidence interval [CI] −12.4, 2.9; *P* = 0.22). Forty-two checklists were excluded from the analysis because they were used on surgical patients admitted to the PICU.

The overall survey response rate was 84%, with 15 (88%) of the 17 PEM physicians and 28 (82%) of the 34 ED nursing staff responding, respectively. Four of the nurses (9%) who participated in the survey were not working in the ED at least 6 months before implementing the BETTER Checklist; their responses were included in all question analyses except questions 5a, 5b, and 5c. All (100%) of the respondents had used the BETTER checklist, with a median of 7 usages (IQR 5, ≥10) overall. There were no significant differences in usage between nurses (median 6.5, IQR 4, 10) and physicians (median 8, IQR 5, 10) (*P* = 0.23).

We achieved our survey’s primary outcome, with 87% and 93% of nurses and physicians, respectively, agreeing that the checklist improves the safety of transporting patients to the PICU (Table 1). Overall, the vast majority of both nurses and physicians agreed that the checklist helped to anticipate potential adverse events and that the use of the checklist should continue in our ED. Almost all nurses and all physicians agreed (or neutral) that the checklist did not contribute to significant delays. There were no statistically significant differences between the median responses of physicians and nurses. Twenty (47%) respondents wrote a comment in the open-ended final question (**Table 1, Supplemental Digital Content 2,** http://links.lww.com/PQ9/A270).

**Table 1.** Results of the Survey on the BETTER Checklist’s Perceived Impacts, Completed by ED Nurses and Physicians 9 Months after Checklist Implementation

Question Number	Strongly Disagree (%)	Disagree (%)	Neutral (%)	Agree (%)	Strongly Agree (%)	r	*P*
1. The BETTER checklist improves the safety of transporting patients to the ICU	RN = 0%	RN = 0%	RN = 13%	RN = 83%	RN = 4%	0.12	0.46
	MD = 0%	MD = 0%	MD = 7%	MD = 40%	MD = 53%		
	All = 0%	All = 0%	All = 10%	All = 67%	All = 23%		
2. The use of the BETTER checklist has helped our team to anticipate potential adverse events that might occur during transport	RN = 0%	RN = 4%	RN = 8%	RN = 83%	RN = 4%	0.11	0.48
	MD = 0%	MD = 0%	MD = 0%	MD = 47%	MD = 53%		
	All = 0%	All = 3%	All = 5%	All = 69%	All = 23%		
3. The ED should continue to use the BETTER checklist for transporting patients to the ICU	RN = 0%	RN = 8%	RN = 21%	RN = 71%	RN = 0%	0.25	0.10
	MD = 0%	MD = 0%	MD = 7%	MD = 33%	MD = 60%		
	All = 0%	All = 5%	All = 15%	All = 57%	All = 23%		
4. The time needed to complete the BETTER checklist does not contribute to significant delays in transporting patients to the ICU	RN = 0%	RN = 8%	RN = 35%	RN = 54%	RN = 13%	0.05	0.75
	MD = 0%	MD = 0%	MD = 0%	MD = 40%	MD = 60%		
	All = 0%	All = 5%	All = 15%	All = 49%	All = 31%		
5a. Since July 2019, the BETTER checklist has Improved communication between ED nurses and physicians for ED patients with an ICU disposition	RN = 0%	RN = 0%	RN = 13%	RN = 79%	RN = 8%	0.27	0.09
	MD = 0%	MD = 0%	MD = 13%	MD = 40%	MD = 47%		
	All = 0%	All = 0%	All = 13%	All = 64%	All = 23%		
5b. Since July 2019, the BETTER checklist has increased the frequency of bedside assessments by ED physicians for patients with an ICU disposition	RN = 0%	RN = 4%	RN = 33%	RN = 46%	RN = 17%	0.39	0.01
	MD = 0%	MD = 0%	MD = 13%	MD = 47%	MD = 40%		
	All = 0%	All = 3%	All = 26%	All = 46%	All = 26%		
5c. Since July 2019, the BETTER checklist has increased the number of medication(s) and/or equipment that we bring with us during transport	RN = 0%	RN = 25%	RN = 25%	RN = 42%	RN = 8%	0.40	0.01
	MD = 0%	MD = 13%	MD = 20%	MD = 53%	MD = 13%		
	All = 0%	All = 21%	All = 23%	All = 46%	All = 10%		

MD, physician; n, number of respondents; *P*, *P* for Spearman correlation test; r, Spearman correlation with survey respondent frequency of use of the BETTER checklist); RN, nurse.

In the survey responses, there was a statistically significant positive correlation between the frequency of using the BETTER checklist and 2 favorable responses: agreement with an increased frequency of bedside assessments by physicians of patients with a PICU disposition (5b), and agreement that an increase in the number of medication(s) and/or equipment were brought on IHT (5c) (Table 1). The rest of the questions had positive but nonsignificant (*P* > 0.05) correlations between the frequency of using the BETTER checklist and the more favorable responses (ie, agreement).

**Fig. 5.**
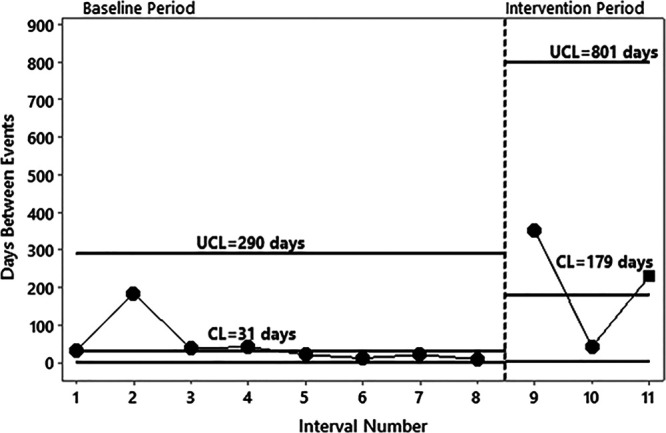
T chart of days between IHT-related issues identified in the hospital’s safety event reporting system for patients admitted from the ED. CL, center line; UCL, upper control limit.

An incident report with an IHT-related issue for ED admissions occurred during 2.3% (9 of 391) and 0.5% (2 of 400) of PICU admissions in the baseline and intervention periods, respectively (difference = 1.8%, 95% CI 0.2, 3.4; *P* = 0.03). Of these 11 reports, the documented issues included medication issues (36%), equipment issues (36%), wrong team member composition (36%), and a clinical instability issue (9%) (**Table 2, Supplemental Digital Content 3,** http://links.lww.com/PQ9/A271). A special cause was noted on the T-chart after the checklist intervention (interval #9) (Fig. 5). There were no differences in the hospital-wide, overall monthly incident report rates during baseline and intervention periods (*P* = 0.16).

## DISCUSSION

This project’s goal was to improve the safety of IHT for ED patients admitted to the PICU. The QI team achieved its primary aims: frontline users sustained a high checklist completion rate and reported improved perceptions of IHT safety with checklist usage. The checklist completion rate (84%) was higher than previously reported IHT checklist completion rates (57%–75%), potentially due to using a process map and audit system.^6,7,14,24^ Although the checklist was an additional task for teams, checklist implementation did not affect the timeliness of objective throughput measures or subjective perceptions of delays, a critical safety issue given that delayed ICU transfers have been associated with higher mortality.^28^

The survey had a high response rate, and most respondents wanted to continue using the checklist, in agreement with a prior survey in which 87% of clinicians wanted an IHT safety checklist.^29^ More frequent use of our checklist was associated with more agreement that the checklist improved patient safety processes, suggesting that users might need multiple uses to realize the checklist’s value. Most of the free-text comments were positive and focused on improvements in anticipating safety issues and situational awareness.^30^ Three respondents suggested that it was a helpful tool but questioned the necessity of the tool “for experienced staff.” Given the nurse–physician dyad approach to using this checklist, frequent staff turnover in the ED environment, and the hospital’s focus on high-reliability safety principles, the QI team promotes consistent checklist usage regardless of staff experience levels.^27^ One limitation of our survey might have been a favorable response bias. We attempted to limit this through an anonymous, voluntary survey design with no incentives.

Incident reporting of IHT-related issues for ED admissions had a relative decrease of 78% after implementing the checklist. Although study design differences limit specific comparisons, this finding is consistent with improvements found in general ED IHT checklist studies associated with relative decreases of 41%–68% in adverse events.^4,6,31^ The reasons for incidents in this study (eg, medication, equipment, team, and clinical instability) were similar to those reported in these other studies. Our care teams assessed for patient stability status and equipment items in ≥95% of the used checklists, which were 2 items associated with decreased harm in other IHT studies and addressed the root causes identified in the original IHT incident from our ED.^6,15^ Although incident reports can be subject to underreporting, the unchanged rate of hospital-wide incident reporting during baseline and intervention periods supports the hypothesis that checklist implementation might have influenced the incidence of these IHT-related events.^32^ None of the QI team physicians (who encompassed 1.5 of the 12.75 full-time-equivalents of clinical shifts staffed) or nurses cared for any patients admitted to the PICU with IHT-related issues during the baseline or implementation periods; an unconscious underreporting bias could have potentially occurred during the implementation period by this small minority of ED physicians. An alternate study design for this project would have been prospective incident data collection from trained IHT observers. Still, concerns for a Hawthorne effect limiting the internal validity of the checklist disallowed such a design.^33^

An unexpected finding was the use of the IHT checklist for PICU admissions that did not meet inclusion criteria as a medical patient. Anecdotal feedback from staff indicated that they used it with surgical and trauma PICU admissions because it promoted team communication, readiness, and situational awareness. In response, the IHT checklist expanded the criteria to include all PICU admissions in October 2020. Other parts of the hospital are also considering adopting the tool for other IHT patient populations. The QI team originally planned to convert the tool from paper to electronic format, but end users have consistently advocated for the continued use of the “index card” format due to its ease of use and portability.^16^

Although previous studies have described the risks of pediatric IHT and adult IHT checklists’ implementation, we are unaware of any studies that have implemented pediatric IHT checklists.^2,4,6–9,21^ Our team demonstrated that using an IHT checklist for pediatric patients admitted to the PICU was feasible to complete in the ED setting. The IHT checklist was associated with perceived improvements in safety and decreased reports of IHT incidents. Although a limitation of this study was its single-center design, the tool could potentially be adopted in other ED settings and evaluated for its generalizability. This description of the structure, design, and implementation of this tool could be an early step in designing pediatric IHT guidelines that could be implemented and validated in a multicenter design.

## DISCLOSURE

The authors have no financial interest to declare in relation to the content of this article.

## ACKNOWLEDGMENTS

We want to acknowledge all ED team members for their openness to changing their behavior to improve patient care. Special thanks to our project champions (Nurses Tracy Grimes, Lateshia Winstead, Becky Roenker Stephanie Graefe, Monica Weeks); Christopher Foley for his input as an ICU stakeholder; Jamie Carter and Jeanette Runner for their help in building the pediatric ICU admission report.

## Supplementary Material


