# Supplementary material for: Implementation of an Intrahospital Transport Checklist for Emergency Department Admissions to Intensive Care
Source: Pediatr Qual Saf. 2021 Jun 23;6(4):e426. doi: 10.1097/pq9.0000000000000426 (PMC8225371; doi:10.1097/pq9.0000000000000426)
Supplement: Supplementary file 1 [file pqs-6-e426-s001.pdf]

Supplemental Digital Content: Figure 1: Survey for users of the Briefing ED-to-ICU Transport To Exit Ready (BETTER) checklist.

BETTER Checklist Survey Questions

This anonymous survey evaluates the BETTER checklist, a tool used by nurses and physicians prior to transferring medical patients to the PICU. Please circle one answer choice for the following questions:

1. The BETTER checklist improves the safety of transporting patients to the ICU.

Strongly Disagree   Disagree   Neutral   Agree   Strongly Agree

2. The use of the BETTER checklist has helped our team to prepare for potential adverse events that might occur during transport.

Strongly Disagree   Disagree   Neutral   Agree   Strongly Agree

3. The ED should continue to use the BETTER checklist for transporting patients to the ICU.

Strongly Disagree   Disagree   Neutral   Agree   Strongly Agree

4. The time needed to complete the BETTER checklist does NOT contribute to *significant* delays in transporting patients to the ICU.

Strongly Disagree   Disagree   Neutral   Agree   Strongly Agree

5. Since July 2019, the BETTER checklist has contributed to the following changes:

   a. Improved communication between ED nurses and physicians for patients with an ICU disposition

      Strongly Disagree   Disagree   Neutral   Agree   Strongly Agree

   b. An increase in the frequency of bedside assessments by ED physicians for patients with an ICU disposition

      Strongly Disagree   Disagree   Neutral   Agree   Strongly Agree

   c. An increase in the number of medication(s) and/or equipment that we bring with us during transport.

      Strongly Disagree   Disagree   Neutral   Agree   Strongly Agree

6. Approximately how many times have you used the BETTER checklist since it was started in July 2019? ________

0   1   2   3   4   5   6   7   8   9   ≥ 10

7. What is your role in the CHKD ED?   NURSE   PHYSICIAN

8. Were you working in the CHKD ED before July 2019?   Yes / No

9. Is there anything that you would change about the current BETTER checklist process?

____

____

Thank you for completing the survey
Please place it inside the manila envelope on the wall in the common staff area.
