# Supplementary material for: Implementation of an Intrahospital Transport Checklist for Emergency Department Admissions to Intensive Care
Source: Pediatr Qual Saf. 2021 Jun 23;6(4):e426. doi: 10.1097/pq9.0000000000000426 (PMC8225371; doi:10.1097/pq9.0000000000000426)
Supplement: Supplementary file 2 [file pqs-6-e426-s002.pdf]

Supplemental Digital Content: Table 1: Qualitative responses to the final question ("Is there anything you would change about the current BETTER checklist process?"), grouped according to survey respondent's answer to question 3.

Survey respondent's answer to: "The ED should continue to use the BETTER checklist for transporting patients to the ICU" (Question 3)	Open-ended response (MD or RN) to: "Is there anything that you would change about the current BETTER checklist process?" (Question 9)
Strongly agree	Nurse: "I have not seen the BETTER checklist used before I got here – I really like it and think it is great for patient safety."<br>Physician: "We are better prepared for adverse events during transport to the ICU."<br>Physician: "It seems fairly comprehensive without being overly burdensome."<br>Physician: "Great checklist that serves as a cognitive team-based aid to anticipate potential safety issues."
Agree	Nurse: "Very useful."<br>Nurse: "It makes people think, which is always good."<br>Nurse: "Make it an online form, maybe in the charting."<br>Nurse: "I think it is the appropriate length needed for the task."<br>Nurse: "It is beneficial if used appropriately but should be common sense for experienced staff – doctors should definitely check with new nurses."<br>Nurse: "Sometimes it is hard to find the attending before transport."<br>Nurse: "If the attending is busy you cannot complete list, causing delays – can the resident or fellow sign off?"<br>Nurse: "Sometimes it's difficult to get the attending to complete if they are in a procedure."<br>Nurse: "Make it a task for the patients admitted in the computer system or something like the code sheet print out."<br>Nurse: "Clarify with docs what seizure meds are needed."<br>Nurse: "Make it an online checklist task like the other transfer of care options."<br>Physician: "I think it works well – much better situational awareness."<br>Physician: "I like having that last reminder to assess the patient before leaving unit."

Neutral	Nurse: “It has been beneficial in the prevention of errors. At the same time, it’s unfortunate that our staff cannot be trusted to think critically without such an elementary system.”<br>Nurse: “I would need to use the tool more to better comment.”
Disagree	Nurse: “I don’t think it is helpful if you have competent staff.”
Strongly disagree	Not applicable (no question 3 respondents with “strongly disagree”)
