# Supplementary material for: Implementation of an Intrahospital Transport Checklist for Emergency Department Admissions to Intensive Care
Source: Pediatr Qual Saf. 2021 Jun 23;6(4):e426. doi: 10.1097/pq9.0000000000000426 (PMC8225371; doi:10.1097/pq9.0000000000000426)
Supplement: Supplementary file 3 [file pqs-6-e426-s003.pdf]

Supplemental Digital Content: Table 2: Hospital safety reporting system events related to intra-hospital transport of patients admitted to the intensive care unit from the emergency department, before (pre) and after (post) the implementation of the intra-hospital transport checklist.

Event Number (Pre- or Post-Intervention)	Severity of Harm Classification (Higher numbers indicate more harm)	Medication Issue	Equipment Issue	Wrong team composition	Clinical Instability
1 (Pre)	1A. Unsafe situation that could contribute to an adverse event		X		
2 (Pre)	4H. Event with harm, near death				X
3 (Pre)	1A. Unsafe situation that could contribute to an adverse event		X		
4 (Pre)	3D.Event without harm, increase monitoring or treatment to prevent harm	X			
5 (Pre)	1A. Unsafe situation that could contribute to an adverse event	X	X		
6 (Pre)	2B2. Near miss. An event occurred but did not reach the patient because of active recovery by caregivers	X		X	
7 (Pre)	1A. Unsafe situation that could contribute to an adverse event			X	
8 (Pre)	1A. Unsafe situation that could contribute to an adverse event		X		
9 (Pre)	1A. Unsafe situation that could contribute to an adverse event			X	
10 (Post)	2B1. Near miss. An event occurred but did not reach the patient because of chance alone.	X			
11 (Post)	1A. Unsafe situation that could contribute to an adverse event			X	

Severity of harm classification according to Chamberlain JM, Shaw KN, Lillis KA, et al. Creating an infrastructure for safety event reporting and analysis in a multicenter pediatric emergency department network. *Pediatr Emerg Care.* 2013;29(2):125-130. Contributing factors to the event are marked with an "X."
